# Initial regional evaluation of the Cystic Fibrosis Newborn Screening Program: data from the Mediterranean coast of Turkey**

**DOI:** 10.3906/sag-1904-198

**Published:** 2019-12-16

**Authors:** Abdurrahman Erdem BAŞARAN, Ayşen BAŞARAN, Dilara Fatma KOCACIK UYGUN, Özgül ALPER, Deniz ACICAN, Ayşen BİNGÖL

**Affiliations:** 1 Department of Pediatrics, Division of Pulmonology, Faculty of Medicine, Akdeniz University, Antalya Turkey; 2 Department of Pediatrics, Division of Pulmonology, University of Health Sciences Antalya Training and Research Hospital, Antalya Turkey; 3 Department of Pediatrics, Division of Allergy Immunology, Faculty of Medicine, Akdeniz University, Antalya Turkey; 4 Department of Medical Biology and Genetics, Faculty of Medicine, Akdeniz University, Antalya Turkey; 5 Department of Child and Adolescent Health, Public Health General Directorate, Ankara Turkey

**Keywords:** Immunoreactive trypsinogen, cystic fibrosis newborn screening, IRT cut-off, Turkey

## Abstract

**Background/ aim:**

Since January 2015, the Cystic Fibrosis Newborn Screening (CFNS) program has been implemented in Turkey. We aimed to evaluate the demographic, clinical, and laboratory data of cases referred from the CFNS program and to determine the most suitable cut-off value for immunoreactive trypsinogen (IRT)-1 and immunoreactive trypsinogen (IRT-2) that are used in the CFNS program in Turkey.

**Materials and methods:**

A total of 156 Turkish Caucasian subjects were determined as positive cases during 3 years, from January 2015 to January 2018, and were referred to the pediatric pulmonology clinics of Akdeniz University Hospital, Antalya, Turkey, for the national CFNS program. The evaluation was made considering the IRT-1 and IRT-2 values, demographic characteristics, sweat test results, CFTR genotypes, and diagnoses.

**Results:**

Nine patients were diagnosed with cystic fibrosis (CF). Eight were diagnosed with CF-related metabolic syndromes and three were determined to be CF carriers. The ratio of CF to CF-related metabolic syndrome was determined as 1.1:1. Considering the limits of the present CFNS program and the IRT method, the positive predictive value (PPV) for the referred cases was determined as 5.8%. When a cut-off value of 105.6 ng/mL was taken for IRT-1, sensitivity was 100%, specificity was 59%, and PPV was 12.8%. For a cut-off value of 88.75 ng/mL for IRT-2, sensitivity was determined as 90%, specificity as 65%, and PPV as 15.2%.

**Conclusion:**

This is the first detailed clinical study to evaluate the data from the CFNS program along the Mediterranean coast of Turkey. As false positive results are extremely high in Turkey, there is an urgent need for revision of the IRT-1 and IRT-2 limits by evaluating the data of the whole country.

## 1. Introduction

Newborn screening programs are important in terms of diagnosing disorders in which intervention before symptoms can improve outcomes. Within the last 50 years, European countries have started newborn screening programs for several hereditary diseases within the framework of public health programs [1,2]. Over the years, cystic fibrosis newborn screening (CFNS) has become the main nucleus of these programs. As the first stage in all CFNS programs, the immunoreactive trypsinogen (IRT) level is measured in blood samples taken in the first week of life. The use of IRT in cystic fibrosis (CF) was first described by Crossley et al. in 1979 [3]. 

Although an increased IRT level in the first week of life is a sensitive marker for determination of infants with CF (OMIM 219700), it is not a specific test. The positive predictive value (PPV) in samples taken on days 2–5 has been reported to be 3%–10% [4]. There is a need for a second test to increase the specificity and reduce the number of referred infants based on the results of the sweat test. As a secondary test, the IRT level is usually remeasured in the 2nd–4th weeks of life, DNA sequencing analysis is performed to assess the presence of CF-causing mutations in hotspot regions, and next-generation DNA sequencing analysis is applied in order to determine any possible mutation in the whole cystic fibrosis transmembrane regulator (*CFTR*; NM_000492) gene [5]. In secondary stage tests, different factors including the geographical structure of each country, phenotypic-genetic heterogeneity, and the accessibility of healthcare resources must be taken into consideration. In countries with high genetic heterogeneity, as is the case for Turkey, generally the IRT level assessment is repeated in weeks 2–4 as the secondary test. 

When the IRT/IRT method is used as the CFNS test, its PPV is approximately 50%. It was reported that perinatal factors such as asphyxial birth, hypoglycemia, or congenital abnormality caused 25% false-positive results in the first IRT level. At the same time, other causes including congenital infection, bowel atresia, renal failure, and some aneuploidies (trisomies 13 and 18) can be influential on false-positive results [4]. False-positive results have also been reported in cases of low birthweight and background of admittance to the neonatal intensive care unit [6].

It is well known that the reporting of the CFNS results of a country makes a significant contribution to the development of screening tests and the control of the disease. Since the CFNS program just recently started in 2015 in Turkey, there is an urgent need for reporting and evaluation of the screening program’s results. Therefore, the main aim of this study was to evaluate the demographic, clinical, and laboratory data of cases referred to our clinics based on the obtained data from the national CFNS program and to determine the most suitable cut-off values for IRT-1 and IRT-2 values, which are used in the CFNS program. 

## 2. Materials and methods

In the CFNS program, which has been implemented in all provinces of Turkey since 2015, heel blood samples are taken on days 3–7 after birth and are examined using the fluorometric enzyme immunoassay method, and cases with IRT levels (IRT-1) of >90 ng/mL are determined. A second IRT level (IRT-2) measurement is then applied on days 14–21 after birth. The newborns with IRT-1 of <90 ng/mL do not give a sample for the second newborn screening and the result is accepted as a complete process. If the IRT-2 level is >70 ng/mL in the cases with high IRT-1, those infants are accepted as CFNS-positive and are referred to CF centers for sweat testing. The patients in the current study determined as CFNS-positive and referred to our center were separated into 2 groups: Group I comprised patients diagnosed with CF as a result of further tests and Group II comprised those who were not diagnosed with CF. 

The data from 156 Turkish Caucasian patients who were determined as CF-positive in the national CFNS program and were referred for sweat testing to the pediatric pulmonology clinics of Akdeniz University Hospital between January 2015 to January 2018 were evaluated. This university hospital is the only CF center in Antalya Province, located on the south coast of Turkey. We obtained written approval from the Clinical Research Ethics Committee of Akdeniz University, Turkey. Data were retrieved in terms of the medical records of the time of presentation, parental consanguinity, birthweight, gestational week at birth, and maternal age. 

The chloride level in the sweat test was measured with the CFΔ Collection System (UCF 2010 Iontophoresis Unit and UCF 2011 Sweat Analysis Unit), which analyzes the Cl− concentration of sweat with Coulometric Endpoint Software [7]. In cases with a chloride (Cl) level of >30 mmol/L in sweat testing, *CTFR* gene testing was performed, and after 1 week, follow-up sweat testing was applied. 

Molecular genetic analysis of *CFTR *gene DNA sequencing was performed using the ABI 3130 Genetic Analyzer (Life Technologies, USA) to assess the presence of CF-causing mutations in hotspot exons. Then next-generation DNA sequencing analysis (Ion Ampliseq CFTR Panel) was applied in order to determine any possible mutations (including deletion and duplication analysis) in the whole cystic fibrosis transmembrane regulator gene (*CFTR*; NM_000492). In next-generation sequencing, CF panel v2 of the Ion AmpliSeq Panel (Thermo Fisher Scientific, USA), which screens the whole gene with 102 amplicons (8.49 kb), was used. Targeted next-generation sequencing analysis was performed (all coding exons, intron-exon boundaries, and UTR regions). Samples were multiplexed and sequenced in an Ion S5 System (Thermo Fisher Scientific, USA). Ion Reporter Software 4.0. (Thermo Fisher Scientific, USA) was used to map the sequence reads to the GRCh37/hg19 reference genome and to call the variants. The Ingenuity Variant Analysis tool (QIAGEN, Germany) was used for annotation and filtering. Variants listed in the CFMDB, CFTR2, dbSNP, 1000G, and ExAC browsers with >1% minor allele frequency were also excluded, and the remaining variants were examined. 

Infants with Cl− levels of >60 mmol/L in the sweat test and/or carrying both mutated *CFTR *alleles were defined as CF-positive, those with persistently intermediate sweat chloride values ranging from 30 to 59 mmol/L and fewer than 2 CF-causing *CFTR* mutations or 2 *CFTR* mutations with 0 or 1 known to be disease-causing and sweat chloride concentration <30 mmol/L were defined as having CF-related metabolic syndrome (CRMS), those with <30 mmol/L in the sweat test and carrying a single mutated allele were defined as CF carriers, and those with wild genotypes were defined as normal [8,9]. 

### 2.1. Statistical analysis

The analysis was conducted using SPSS 23.0 (IBM Corp., Armonk, NY, USA). Descriptive statistics were presented as percentage, mean, standard deviation, median, minimum, and maximum values. To test the difference of the numeric variables between groups, the Student t-test or Mann–Whitney U test was used. To test the difference of the categorical variables between the groups, the chi-square test was used. Receiver operating characteristic curve (ROC) analysis was used for determining the best cut-off value of IRT-1 and IRT-2. A significance level of 95% (or error margin of α = 0.05) was used for determining the differences in analyses. 

## 3. Results

From 2015 to 2018, 104,255 live births in Antalya Province, southwest Turkey, were recorded. The newborn screening test was performed for all of these babies. In the first IRT screening of this cohort, 1738 newborn babies were found with IRT-1 levels of >90 ng/mL. In the second test, the IRT levels of 156 babies were above 70 ng/mL (IRT-2 > 70 ng/mL) and they were referred to our CF center for further clinical evaluation. Although the CFNS test result was negative (IRT-1 level was 270 ng/mL, IRT-2 level was 23 ng/mL), one patient with chronic *Pseudomonas aeruginosa* colonization and respiratory problems was diagnosed with CF. The result of sweat testing for this patient was 28.1 mmol/L and the *CFTR* genotype was determined as compound heterozygous, p.R668C/p.G576A. 

The 156 Turkish Caucasian patients included in the study comprised 92 (59%) females and 64 (41%) males. There was a history of parental consanguinity in 31 (19.9%) cases. Based on these data, 9 patients were diagnosed with CF, 8 with CF-related metabolic syndrome, and 3 as CF carriers. The ratio of CF to CF-related metabolic syndrome was 1.1:1. The demographic characteristics, the results of the sweat testing, and the *CFTR* genotypes of these 20 Turkish Caucasian patients are shown in Table 1. 

**Table 1:** The demographic characteristics, sweat test results, and CFTR genotypes of the diagnosed patients.

Case	Diagnosis	Sex(F/M)	Time of presentation (days)	IRT-1(ng/mL)	IRT-2(ng/mL)	Cl in sweat (mmol/L)	CFTR genotype
1	CF*	F	50	171	89	86	c.621+1G>T/[?]
2	CF	M	110	139	156	65	p.ÄF508/p.S549R
3	CF	M	120	110	128	69	p.G1244V/p.G1244 V
4	CF	F	26	303	176	89	p.N1303K/p.N1303K
5	CF	F	34	116	121	47	c.2789+5G>A/ c.2789+5G>A
6	CF	F	63	204	372	79	p.L732X/p.L732X
7	CF	M	69	252	210	108	[?]/[?]
8	CF	F	64	106	93	70	c.2789+5G>A/ c.2789+5G>A
9	CF	M	37	118	131	70	c.406-1G>A/ c.2184insA
10	CF carrier€	M	34	98	171	13	p.K68E/ [?]
11	CF carrier	F	40	162	70	9	p.V920L/[?]
12	CF carrier	F	24	169	224	10	p.E217G / [?]
13	CRMS£	F	29	142	124	35	[? ]/ [?]
14	CRMS	F	34	160	116	44	p.Y301C/[?]
15	CRMS	M	58	90	90	42	p.L997F/[?]
16	CRMS	M	44	101	70	37	[? ]/ [?]
17	CRMS	F	38	90	70	39	[? ]/ [?]
18	CRMS	M	30	122	70	33	[? ]/ [?]
19	CRMS	M	31	129	86	49	p.F1052V/ [?]
20	CRMS	M	30	125	70	38	p.L997F / [?]

*CF: Cystic fibrosis. €CF carrier: Cystic fibrosis carrier. £CRMS: Cystic fibrosis-related metabolic syndrome. IRT-1: First immunoreactive trypsinogen level. IRT-2: Second immunoreactive trypsinogen level. F: Female. M: Male. [?] : Unknown.

The mean time of presentation was 41.44 ± 22.86 days (ranging from 18 to 172 days). The mean of IRT-1 was determined as 115.82 ± 35.9 ng/mL (ranging from 90 to 302.9 ng/mL), the mean of IRT-2 was found as 91.98 ± 35.31 ng/mL (ranging from 70 to 372 ng/mL), the mean amount of Cl in the sweat was found as 19.06 ± 17.02 mmol/L (ranging from 10 to 108.3 mmol/L), the mean birthweight was 3162.98 ± 532.42 g (ranging from 1600 to 4500 g), the mean gestational week at birth was 38.71 ± 1.88 weeks (ranging from 29 to 41 weeks), and the mean maternal age was 28.85 ± 6.29 years (ranging from 18 to 46 years). When the patients were divided between those diagnosed with CF (Group I) and those not diagnosed with CF (Group II), no statistically significant difference was determined between the two groups with respect to gender, parental consanguinity, and birthweight. In Group I, a later time of presentation (63.67 ± 32.68 days vs. 40.08 ± 21.53 days; P = 0.01), higher IRT-1 level (168.79 ± 70.59 ng/mL vs. 112.58 ± 30.2 ng/mL; P = 0.001), and higher IRT-2 level (163.93 ± 86.95 ng/mL vs. 87.57 ± 23.89 ng/mL; P<0.001) were striking, while the gestational week at birth was lower (36.56 ± 3.32 weeks vs. 38.84 ± 1.69 weeks; P = 0.007) (Table 2).

**Table 2:** Comparison of the clinical characteristics of Group I and Group II#.

	Group I	Group II	P-value
Sex (%)	44.4%	40.8%	1.00
Consanguinity (% present)	44.4%	18.4%	0.07
Birthweight (g)*	3031.11 ± 617.61	3171.05 ± 528.12	0.62
Time of presentation (days)*	63.67 ± 32.68	40.08 ± 21.53	0.01
IRT-1 (ng/mL)*	168.79 ± 70.54	112.58 ± 30.2	0.001
IRT-2 (ng/mL)*	163.93 ± 86.95	87.57 ± 23.89	<0.001
Gestational week at birth (weeks)*	36.56 ± 3.32	38.84 ± 1.69	0.007

*Values are stated as mean ± SD. #Group I: Patients diagnosed with CF as a result of further tests; Group II: those not diagnosed with CF.

As shown in the Figure, the areas under the ROC curve (AUC) for IRT-1 and IRT-2 in the discrimination of cystic fibrosis vs. no cystic fibrosis in CFNS were 0.825 (95% confidence interval [CI]: 0.715–0.935) and 0.903 (95% CI: 0.822–0.984) (Figure). When a cut-off value of 105.6 ng/mL was taken for IRT-1, sensitivity was 100%, specificity was 59%, and PPV was 12.8%. When a cut-off value of 88.75 ng/mL was taken for IRT-2, sensitivity was determined as 90%, specificity 65%, and PPV as 15.2%. Considering the limits of the existing cystic fibrosis newborn screening program, the PPV of referred cases to our hospital was determined as 5.8%. 

**Figure:**
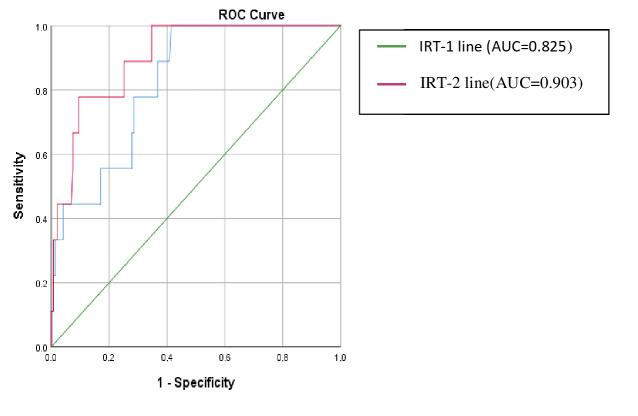
Receiver operating characteristic curve (ROC) for IRT-1 and IRT-2.

## 4. Discussion

It is well known that cystic fibrosis is an autosomal recessive inherited metabolic disorder, which results from a mutation in the transmembrane conductor regulator gene and has been included in the Newborn Screening Program in Turkey since January 2015. To date, the frequency of cystic fibrosis has been reported as 1/3000 in Turkey in limited studies; however, it is thought that this ratio is actually higher due to the consanguinity across the country [10,11]. This study can be considered valuable as it is the first regional cohort that has evaluated the data of the CFNS program from the Mediterranean coast of Turkey. As a result of the study, it was revealed that the IRT-1 and IRT-2 values of the CFNS program are higher in CF cases. We tried to determine the PPV value of the referred cases. After that, the limits of the existing screening program were taken into consideration and the PPV of cases referred to our hospital was determined as 5.8%.

In the European Cystic Fibrosis Association Standards of Best Practice Guidelines, it is recommended that national screening programs must aim for a PPV of at least 30% and sensitivity of 95% [12]. In a study by Barben et al. [13], the national CFNS programs of sixteen European countries since 2016 were examined. They found that ten programs (Northern Ireland, Austria, France, Russia, Slovakia, Czech Republic, the Netherlands, Norway, Turkey, and Portugal) used a fixed cut-off for IRT-1 that ranged from 60 to 90 ng/mL (median: 65 ng/mL). Six programs (Wales, Scotland, England, Poland, Ireland, and Switzerland) had a floating cut-off to achieve a set percentage of samples sent for the next level of testing (ranging from the 99.0th to 99.5th percentiles). Four programs (Wales, France, Czech Republic, and the Netherlands) did not achieve the minimum of 95% standard for sensitivity, and five programs (Northern Ireland, Austria, Slovakia, Czech Republic, and Wales) had a PPV that was lower than the minimum standard of 30%. Ten countries employed a strategy called a ‘safety net’ (also known as ultra-high IRT). In this strategy, patients whose first IRT value was above the determined high value were referred for advanced tests even if the second-step tests were normal. Ultra-high IRT cut-off values were 170 ng/mL in Wales, 100 ng/mL in France, 200 ng/mL in the Czech Republic, 100 ng/mL in the Netherlands, and 400 ng/mL in Norway. In order to consider these criteria in the National Cystic Fibrosis Newborn Screening Program in Turkey, which has extremely high false-positive results, it is necessary to determine new IRT-1 and IRT-2 limits after evaluating all the national data. We believe that employing ultra-high IRT cut-off values can improve the sensitivity of CFNS tests based on our missed case results. In this context, the current study can be considered as guidance, as when a cut-off value of 105.6 ng/mL was considered for IRT-1, sensitivity was 100%, specificity was 59%, and PPV was 12.8%, and when a cut-off value of 88.75 ng/mL was considered for IRT-2, sensitivity was determined as 90%, specificity as 65%, and PPV as 15.2%.

In the European Cystic Fibrosis Association Standards of Best Practice Guidelines, it is stated that after CFNS positivity observation, patients should be seen by a CF specialist team at mean of 35 days and no later than 58 days [12]. In accordance with these criteria, all the patients in the current study presented at a mean of 41.44 ± 22.86 days (varying between 18 and 172 days). However, the mean time of presentation was significantly higher among the patients diagnosed with CF compared to those not diagnosed with CF (63.67 ± 32.68 days vs. 40.08 ± 21.53 days; P = 0.01). This discordance can be attributed to different factors; some patients first presented to another center because the patients were symptomatic, and differences exist in sociocultural levels, delay in taking the second sample, and delay in the transportation of the sample. 

The gestational week at birth in the patients diagnosed with CF was determined to be significantly lower than that patients not diagnosed with CF (36.56 ± 3.32 weeks vs. 38.84 ± 1.69 weeks; P = 0.007). In addition, the mean birthweight of the patients diagnosed with CF was 140 g lower compared to those not diagnosed with CF (3031.11 ± 617.61 g vs. 3171.05 ± 528.12 g). In 2018, Schlüter et al. examined the effect of CF on birthweight in Danish and Welsh populations. The birthweight of CF infants was a mean of 200 g lower and the birth week was a mean of 1 week earlier compared to infants not diagnosed with CF [14]. These results demonstrated the effect of CF disease on the gestational week of birth and birthweight, which must be taken into consideration. 

In a study by Barben et al. [13] examining the national CFNS programs of thirteen European countries since 2014, the CF to CF-related metabolic syndrome diagnosis ratio was reported to vary between 32:1 and 1.2:1. Some countries use DNA analysis of heel blood samples as a second-line test after IRT-1 to shorten the time of diagnosis and increase the PPV. This increases the number of patients diagnosed as cystic fibrosis carriers and with CF-related metabolic syndrome. In the current study, this ratio was determined as 1.1:1, which is lower than in all the other countries. This outcome may be reasonable because the diagnostic criteria for CF-related metabolic syndrome/“CF Screen Positive and Inconclusive Diagnosis” in Europe have only been clearly determined since 2015 [15], and we think that the data collected in the previous period may not have considered these diagnostic criteria, particularly in countries in which screening programs were started earlier than in Turkey. Groves et al. [16] reported that 48% of patients with CF-related metabolic syndrome diagnosis could be later diagnosed with CF within 15 years. It must be taken into account that the number of CF-diagnosed patients in our study population could increase based on the collection of whole DNA sequencing analysis data all throughout the country.

Overall, there are several screening protocols and algorithms throughout the world. In 2016, Barben et al. described sixteen different approaches in 16 European countries [13]. It was concluded that there is no single ideal screening program method for any country. However, in light of the data obtained from the CFNS program of any country, the IRT cut-off values, and the type of* CFTR *gene mutations, a screening method can be developed. Since the identification of the *CFTR *gene in 1989, more than 2065 mutations have been identified throughout the gene to date [17,18]. It is well known that the spectrum of *CFTR *mutations varies among different populations, ethnic backgrounds, and geographical locations [19]. Thus, it is clear that there is no “one-size-fits-all” universal mutation panel for CF testing in different populations. Despite the comprehensive screening of the entire *CFTR *coding regions, the mutations identified do not account for 100% of the molecular defects in CF patients. This may be due to several reasons; there might be mutations deep in introns that are not analyzed, deletions of exons that are not PCR-amplified, genetic variations or mutations in *CFTR *or other genes adversely affecting CFTR function, or a CF phenocopy that has the CF clinical phenotype without mutations in the *CFTR *gene [20,21]. 

In our opinion, the reasons why CFNS false positivity is high in Turkey include the use of fixed cut-off values for IRT-1 and IRT-2 and the lack of an appropriate cut-off value, and also the lack of DNA sequencing analysis in the CFNS program. In a previous study [13], Scotland (sensitivity 100%, PPV 75%) and Ireland (sensitivity 100%, PPV 44%) had the highest sensitivity and positive predictive values. These two countries use the IRT-DNA-IRT method. For IRT-1, a 99.5% floating cut-off is used in Scotland and a 62 ng/mL fixed cut-off is used in Northern Ireland. In 2009, Sontag et al. [22] also reported that the IRT/IRT/DNA model increases the sensitivity of the screening test and reduces the amount of sweat testing in Colorado compared with the IRT/IRT model.

 In conclusion, screening programs are extremely important for the early diagnosis of life-changing diseases, especially for autosomal recessive inherited fatal genetic diseases. In 2017, the Turkish Statistical Institute revealed a cross-sectional and national-level study in 81 provinces that detected a high frequency (18.5% to 57.8%) of consanguinity both in rural and urban areas in the Turkish population. There is a high level of consanguineous marriages all over the country, such as 33.9% on the Mediterranean coast [10], and the rate was found to be 22%–24% with a resistance to reduction by Koç et al. in 2017 [11]. Therefore, the efficacy of screening programs must be carefully evaluated based on the sociodemographic structure and important risk factors for each population. Our expert clinical unit is the only CF center in Antalya Province, on the Mediterranean coast of Turkey, and has been functioning since 1990. We are aware that heterogeneous *CFTR *mutations can cause unusual electrophysiological or clinical manifestations. Based on our cohort, there was only one diagnosed case that could not be determined with the CFNS test. However, although this is an indication of the high sensitivity of the screening program, high false-positive values ​​indicate the need for urgently determining new IRT cut-off values.
